# Phage–bacterial contig association prediction with a convolutional neural network

**DOI:** 10.1093/bioinformatics/btac239

**Published:** 2022-06-27

**Authors:** Tianqi Tang, Shengwei Hou, Jed A Fuhrman, Fengzhu Sun

**Affiliations:** Department of Quantitative and Computational Biology, University of Southern California, Los Angeles, CA 90089, USA; Department of Ocean Science and Engineering, Southern University of Science and Technology, Shenzhen 518055, China; Marine and Environmental Biology, Department of Biological Sciences, University of Southern California, Los Angeles, CA 90089, USA; Marine and Environmental Biology, Department of Biological Sciences, University of Southern California, Los Angeles, CA 90089, USA; Department of Quantitative and Computational Biology, University of Southern California, Los Angeles, CA 90089, USA

## Abstract

**Motivation:**

Phage–host associations play important roles in microbial communities. But in natural communities, as opposed to culture-based lab studies where phages are discovered and characterized metagenomically, their hosts are generally not known. Several programs have been developed for predicting which phage infects which host based on various sequence similarity measures or machine learning approaches. These are often based on whole viral and host genomes, but in metagenomics-based studies, we rarely have whole genomes but rather must rely on contigs that are sometimes as short as hundreds of bp long. Therefore, we need programs that predict hosts of phage contigs on the basis of these short contigs. Although most existing programs can be applied to metagenomic datasets for these predictions, their accuracies are generally low. Here, we develop ContigNet, a convolutional neural network-based model capable of predicting phage–host matches based on relatively short contigs, and compare it to previously published VirHostMatcher (VHM) and WIsH.

**Results:**

On the validation set, ContigNet achieves 72–85% area under the receiver operating characteristic curve (AUROC) scores, compared to the maximum of 68% by VHM or WIsH for contigs of lengths between 200 bps to 50 kbps. We also apply the model to the Metagenomic Gut Virus (MGV) catalogue, a dataset containing a wide range of draft genomes from metagenomic samples and achieve 60–70% AUROC scores compared to that of VHM and WIsH of 52%. Surprisingly, ContigNet can also be used to predict plasmid-host contig associations with high accuracy, indicating a similar genetic exchange between mobile genetic elements and their hosts.

**Availability and implementation:**

The source code of ContigNet and related datasets can be downloaded from https://github.com/tianqitang1/ContigNet.

## 1 Introduction

Mobile genetic elements (MGEs) are abundant in natural environments. They move around in the biosphere and transfer from one host to another to replicate. Among various classes of MGEs, viruses that infect bacteria referred as bacteriophages or phages in short have played essential rules in natural environments. The vital activities and replication of phages require the infection of phages to their hosts. Therefore, it is important to understand the association relationship between phages and their hosts. Several computational methods have been developed to assign viruses to their putative hosts (Ahlgren *et al.*, 2017; Amgarten *et al.*, 2020; Coutinho *et al.*, 2021; Galiez *et al.*, 2017; Lu *et al.*, 2021; Pons *et al.*, 2021; Shang and Sun, 2021; Tan *et al.*, 2022; Wang *et al.*, 2020), most of which assign phages to their hosts of known bacterial genomes or at certain taxonomic levels (Amgarten *et al.*, 2020; Coutinho *et al.*, 2021; Pons *et al.*, 2021; Shang and Sun, 2021; Tan *et al.*, 2022; Wang *et al.*, 2020) except VirHostMatcher (VHM) (Ahlgren *et al.*, 2017) and WIsH (Galiez *et al.*, 2017).

On the other hand, genomes of novel uncultured microbes are usually incomplete due to limitations of shot-gun sequencing. Contigs of novel genomes recovered from metagenomes, and novel genomic islands of known microbes, are absent or incomplete in current reference databases, preventing host predictions using alignment-based methods. Similarly, most viral genomes recovered from viromes are fragmented, making the whole viral genome-based host prediction impractical.

The most widely used methods for phage–host association prediction are based on oligonucleotide frequency, alignment-based scores, matching of CRISPR spacers or Markov models. For example, VirHostMatcher (Ahlgren *et al.*, 2017) uses the dissimilarity between oligonucleotide frequencies of the phage and bacterial sequences to predict their associations. The authors investigated the prediction accuracy based on Euclidean distance, Manhattan distance, and $d_{2}^{*}$ dissimilarity (Reinert *et al.*, 2009; Song *et al.*, 2013; Wan *et al.*, 2010) and showed that $d_{2}^{*}$ yielded the highest prediction accuracy (Ahlgren *et al.*, 2017). WIsH (Galiez *et al.*, 2017) is another tool that trained a Markov model for each candidate bacterial genome and calculated the likelihood of a phage sequence under the trained Markov model. This method was also reported to have better performance than $d_{2}^{*}$ when fragments were used in the prediction (Galiez *et al.*, 2017). VirHostMatcher-Net (Wang *et al.*, 2020) uses logistic regression to take advantage of multiple dissimilarity measures, including the aforementioned $d_{2}^{*}$ and WIsH scores, alignment-based scores, CRISPR spacer matching and virus similarity to predict phage–host associations. RaFAH (Coutinho *et al.*, 2021) is another recently developed method for phage–host association prediction by constructing hidden Markov models (HMMs) according to the protein clusters identified from the pVOG (Grazziotin *et al.*, 2017) database and a random forests model was used to predict phage–host associations.

Deep learning-based methods were also developed in recent years for predicting phage–host associations. vHULK (Amgarten *et al.*, 2020) applied deep neural networks to the phage–host prediction problem using features extracted from phage sequences as input. The latest proposed method HostG (Shang and Sun, 2021) used a graph convolutional networks to take both phage–host and phage–phage relationships into consideration, and the alignment scores were used to aid the construction of the relationships. However, these predefined features extracted from sequences alone are not suitable to train complicated models to predict contig–contig relationship.

In this article, we propose ContigNet, a convolutional neural network (CNN)-based model that can predict the association status between phage and bacterial contigs, the first method investigating the relationship between two contigs. Our results showed that ContigNet was able to significantly improve the phage–host contig association prediction performance compared to currently available methods. Based on the structure of ContigNet, we also tested it on a totally different plasmid-host dataset, and the result revealed its capability as a promising approach for predicting general MGEs associated with their hosts.

## 2 Materials and methods

### 2.1 Dataset preparation

**Fig. 1.**
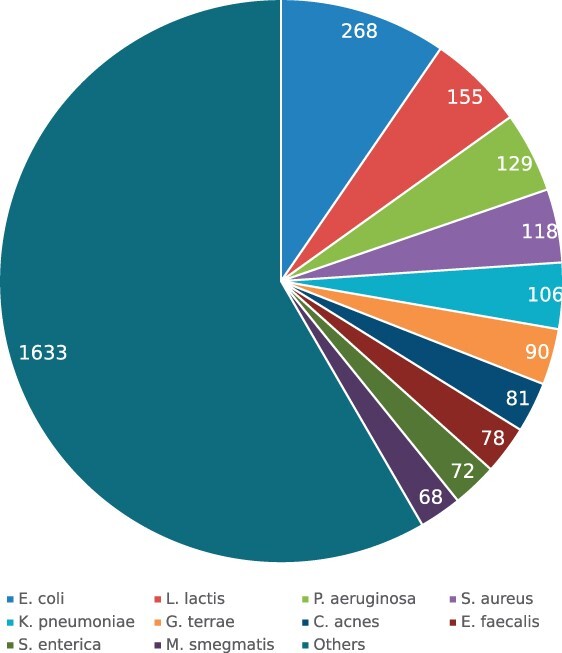
Pie chart showing the number of phages having association with a host species in Virus-Host DB. Top 10 species are shown in the pie chart and the remaining species are combined as ‘Others’

The first dataset, we used was retrieved from Virus-Host DB (Mihara *et al.*, 2016). Virus-Host DB gathered virus and host information from multiple sources, including RefSeq, GenBank, UniProt, ViralZone and manual curation. The association between virus and host was represented as a pair of virus genome sequence and host taxonomic ID in the database. The Virus-Host DB version used in this article was released in March 2021, and this release contained 16 048 virus-host pairs with both prokaryotic and eukaryotic hosts. We filtered out non-bacterial host entries from the database. The hosts reported in the database varied in taxonomic ranks, and only phage–host pairs with host taxonomy ID at species rank were used. We then removed phage–host pairs with host taxonomy ID not presented in GenBank (Benson *et al.*, 2012). Finally, we got a dataset with 2589 phage–host pairs, including 2548 phages and 301 host species. The species level composition of the hosts of the phages is shown in Figure 1 that shows the top 10 most abundant species and the others. From the pie chart, we can see the hosts of the phages are not overly biased toward particular species.

The dataset was separated into training and validation sets. To prevent extremely similar phage genomes appearing in both the training and validation sets, we used CD-Hit v4.8.1 (Fu *et al.*, 2012) to cluster phage genomes, which required >95% sequence identity and >50% alignment coverage for the shorter sequence. Finally, we obtained 2325 phage clusters and in each cluster, we randomly selected a phage chromosome to represent the sequence of other phages in the same cluster. After redundant phage genomes were removed, the dataset was randomly separated into 80% training set and 20% validation set. Any phage–host pair in the training set with the phage also appearing in the validation set was moved to the validation set. A similar approach was also applied to hosts. Given a particular taxonomy level, phage–host pairs with overlapping hosts between the training and validation sets were moved to the validation set.

Virus-Host DB included genome sequences for all viruses in the database, and their host genome sequences were retrieved from GenBank according to their species taxonomy IDs. Some host species, such as *Escherichia coli*, have over 20 000 assemblies in GenBank, and it is not feasible to use all of them for training and testing. Thus, for hosts with more than 10 assemblies, we randomly downloaded 10 assemblies as representatives of that species. For host species with fewer than 10 assemblies, we downloaded all available assemblies. All collected genome sequences were used in the subsequent training procedures.

To assess the generalization ability of our developed model, we also included two different datasets as test sets in our study. The first one is the Metagenomic Gut Virus (MGV) (Nayfach *et al.*, 2021), and the other is the PLSDB (Galata *et al.*, 2019) database. Both datasets were preprocessed so that they did not overlap with the training set. The details of the datasets, steps of preprocessing and rationale of choosing these two datasets as test sets will be discussed in Section 3.6.

### 2.2 Feature representation

For a phage contig with length *L_p_*, we used a one-hot matrix to represent the contig. Particularly, A, C, G and T were represented by [1,0,0,0],[0,1,0,0],[0,0,1,0] and [0,0,0,1], respectively. We used [0,0,0,0] to represent bases other than A, C, G or T that might appear in the sequence. The constructed matrix was denoted as *M_pb_*, with size $L_{p}\times 4$, where the subscript *p* represents the phage and *b* represents nucleotide base. Similarly, for the host, we constructed the one-hot matrix *M_hb_* with size $L_{h}\times 4$, where *L_h_* represents the host contig length.

We also encoded the contigs based on codons using a 64 dimensional one-hot matrix, given that there are 64 possible codons, and a vector with all components being 0 was used in case the number of remaining nucleotides were not enough to form a codon or a non-regular character occurs in the sequence. Contigs were translated into peptides using 3-frame encoding, using the forward strand. Considering a fragment ATGCGTCAT, the possible reading frames can be ATG/CGT/CAT, TGC/GTC/AT-, or GCG/TCA/T-. We first constructed a one-hot matrix for each reading frame and got three individual matrices. The three matrices were concatenated together and used as the input of ContigNet. We used *M_pc_* and *M_hc_* to denote the resulting matrices for phage and host, respectively, where the subscript *c* indicates codon information for the phage or the host.

### 2.3 Deep learning model structure and training

**Fig. 2.**
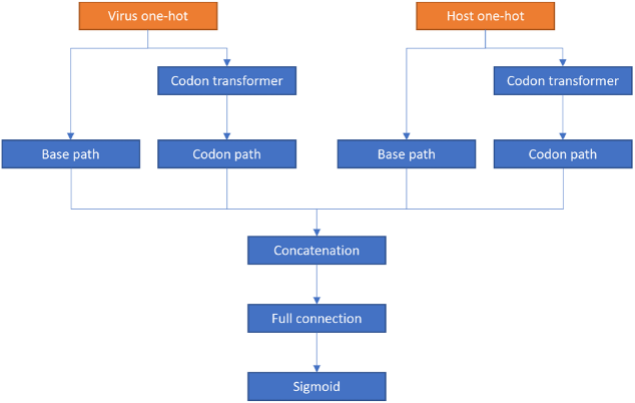
The overview of the deep learning model

The ContigNet is a four-path CNN for the phage–host prediction task. The overview of the model is depicted in Figure 2. It takes matrices *M_pb_* and *M_hb_* as input and outputs a single numeric value indicating the probability if the query phage and host are associated with each other. Each of the two input matrices is branched into two paths, a base-path to encode base information and a codon-path to encode the codon information contained in the contig. In the codon-path, the codon one-hot matrices *M_pc_* and *M_hc_* can be converted from base matrices with a series of convolutional operations, which is named as codon transformer in the figure.

The codon transformer first constructs a filter *F* with size 64×3×4, i.e. the filter consists of 64 kernels, where each kernel is a 3 × 4 matrix. Here we set each kernel to be a base one-hot matrix of a possible codon. Consider a base one-hot matrix *M* with size 3 × 4, representing a 3-mer. The following function

f(M)=ReLU(F*M−2×1)

where the output of * is defined as $[\sum_{i}\sum_{j}F_{0,i,j}\times M_{i,j},\ldots ,\sum_{i}\sum_{j}F_{64,i,j}\times M_{i,j}],\;1$ denotes the vector with all dimensions as 1 and the *ReLU* denotes the Rectified Linear Unit. The output of *f* is a one-hot vector, where the dimension corresponding to the codon is 1 and all other dimensions are 0. By applying the convolutional filter to the base one-hot matrix, we can obtain the corresponding codon one-hot matrix.

The structure of the base and codon channels consists of convolutional and max pooling layers with ReLU activation function. Usually, CNNs require a fixed size input. However, in metagenomics studies, the contig length can range from only a few hundred bps to over thousands of bps. To solve the incompatibility between variable contig length and fixed CNN input size, in ContigNet, we took advantage of the characteristic of the pooling layer to enable its ability to accept a theoretically unlimited length of contigs. The last layer of each convolution channel in ContigNet is a global pooling layer, which produces a fixed length output that can be fed into the downstream fully connected layers, regardless of the length of input. We forced the base paths and codon paths between the phage and host to have the same weight, the rationale will be discussed in Section 3.1.

The outputs of four separated paths are collected, concatenated, and fed into the fully connected layer with sigmoid function as the final output of the model, which has a range of (0, 1), indicating the probability of the two input contigs of being associated with each other.

The model was optimized by minimizing the binary cross-entropy loss between the output and the target. The hyper-parameters for the model training were chosen from grid search using ten-fold cross-validation on the training set. The learning rate was chosen from 0.01, 0.001, 0.0001 and 0.00001, and the batch size was chosen from 16, 32, 64 and 128. Eventually, the model was trained for 5000 epochs with early stopping criteria 0.00001 for 50 epochs. The learning rate was finally set to 0.001 and the batch size was set to 32.

At each epoch, for a positive phage–host pair, we need to construct a negative phage–host pair. In this study, we randomly selected the host that was not reported interacting with the phage in Virus-Host DB to construct the negative phage–host pairs. This way of selecting the negative pairs can possibly include some phage–host pairs that have associations but not reported by previous studies. However, such potential positive phage–host pairs not reported in the database are expected to be extremely rare given that the number of truly associated phage–host pairs is much smaller than that of negatively associated pairs. Thus, we expect that such incorrectly chosen pairs will have minimal impacts on our model. For each positive phage–host pair, we constructed one negative phage–host pair resulting in the positive/negative ratio of 1:1.

### 2.4 Investigation of the effects of contig lengths, sequencing errors and chimeric contigs on the performance of ContigNet

To investigate the effects of contig lengths on prediction accuracy, for each input batch during training, validation, and testing, the phage contig length *L_p_* was selected from 200 bps, 500 bps, 1 kbps, 5 kbps, 10 kbps and 50 kbps, and the host contig length *L_h_* was selected randomly from 200 bps, 500 bps, 1 kbps, 5 kbps, 10 kbps, 50 kbps and 100 kbps. Contigs were then sampled from the corresponding phage and host genomes. The contig lengths and contig sequences were all re-sampled at the start of each training epoch to increase the diversity of the training dataset. Firstly, we evaluated the performance of the trained model on phage–host contig pairs directly sampled as error-free substrings of phage and host genomes. The related results were reported in Sections 3.2 and 3.3.

In metagenomic samples, however, it is possible that the contigs have multiple sources of errors including sequencing and assembly errors. Therefore, we also assessed the performance of ContigNet with different types of errors. For sequencing, possible errors include insertions, deletions and substitutions. The error profiles can vary depending on sequencing (NGS) technologies, and most common sequencing technologies have error rate <0.1% (Ma *et al.*, 2019). To simulate the general scenario, we set two parameters, *μ* as substitution rate and *δ* as insertion/deletion rate. For each nucleotide, given an error occurred, a substitution error occurred with probability $\frac{\mu }{\mu +\delta }$ and the nucleotide was changed to the other nucleotides with equal probability $\frac{1}{3}$. A deletion error occurred with probability $\frac{\delta }{2(\mu +\delta )}$. An insertion error occurred with probability $\frac{\delta }{2(\mu +\delta )}$ and one of A, C, G or T was inserted with equal probability $\frac{1}{4}$. We used this simple model to show the impact of substitution and insertion/deletion errors on the performance of ContigNet. Other sequencing error mechanisms can easily be simulated. In this experiment, we first trained ContigNet with error-free training set, and the trained model was evaluated on the validation set with artificially simulated errors with different combinations of *δ* and *μ*. The parameter *δ* was chosen from 0, 0.05 and 0.1, and *μ* was chosen from 0 to 0.1 by step of 0.02. The parameters for the simulations could be over 100 times larger than typical error rates, and therefore the results showed the lower bound of the performance of ContigNet on real metagenomic datasets. The contig lengths for both phage and host were fixed at 5 kbps, and the area under the receiver operating characteristic curve (AUROC) of ContigNet on the test sets was reported as the average of 10 repeated experiments.

There are potential assembly errors of the contigs in metagenomic studies. Reads from different genomes could be assembled into the same contigs referred as chimeric contigs. We assessed the effect of the presence of chimeric contigs in the test set on the performance of error-free trained ContigNet. To simulate the occurrence of chimeric contigs, for each phage/host contig, we exchanged 0–20% of its sequence with a random contig in the validation set. We only swapped the subsequences between phages and between hosts, because previous studies showed that it is very rare to mix viral and host fragments in assembly (Magasin and Gerloff, 2015; Pignatelli and Moya, 2011). The AUROC scores of ContigNet on the validation set with sequencing errors and chimeric contigs were reported in Section 3.5.

### 2.5 Performance evaluation

The performance of our deep learning method in the phage–host association prediction was assessed using the AUROC score. The receiver operating characteristic (ROC) curve visualizes the predictive performance of a binary classifier by plotting the true positive rate against the false positive rate at various thresholds. The AUROC can be used to quantify the performance of this binary classifier.

## 3 Results

### 3.1 Sharing weights among base and codon paths improves model generalization

The convolutional part of ContigNet is essentially a feature extractor, where each path extracts 256 features from the base and codon paths of the input sequence. Comparing with traditional alignment-free-based methods, we can consider the output of the convolution layer as the *k*-mer frequencies, and the fully-connected layers as the distance measure. When we calculate *k*-mer frequencies, we treat phage and host sequences the same and identical features are extracted from them. Hence, it is also reasonable to let the convolutional paths between both sequence types to have the same weights.

To legitimize the use of shared weights, we need to compare two models, one having shared weight between phage and host paths and the other having independent weights between phage and host paths. For a fair comparison, for both models, we conducted a grid search using 10-fold cross-validation on the training set, with the learning rate chosen from 0.01, 0.001, 0.0001 and 0.00001, and the batch size chosen from 16, 32, 64 and 128. The final selected hyperparameters were the same for both models, with the learning rate being 0.0001 and batch size being 32.

**Fig. 3.**
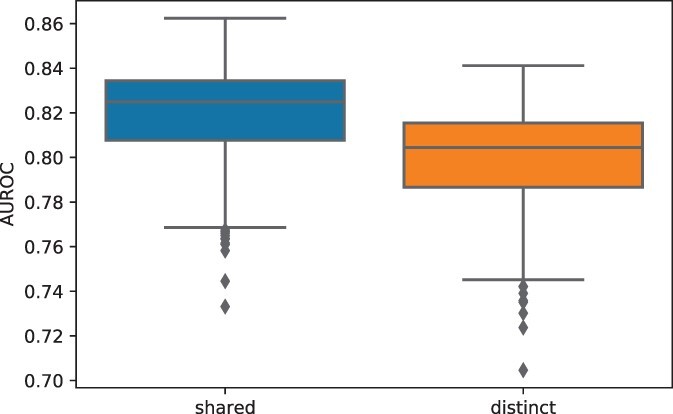
ContigNet with shared weights outperforms ContigNet with distinct weights. Boxplots comparing AUROC scores of ContigNet models with and without shared weights between phage and host paths

The trained models were then tested on the validation set. The AUROC scores of each model on contigs with different lengths, ranging from 200 bps to 5 kbps, are shown in Figure 3. The figure shows that ContigNet with shared weights for phages and hosts outperforms the model with independent weights. The Wilcoxon signed-rank test also supports this conclusion with *p*-value $1.33\times 10^{-25}$. Therefore, in the rest of the article, we use the model with shared weights between the phages and the hosts.

### 3.2 Contignet increased the prediction performance compared to existing methods

K-mer-based alignment-free sequence comparison methods have been widely used for predicting phage–host associations. Among various methods of the same class, the currently known best performing alignment-free method is $d_{2}^{*}$ dissimilarity (Ahlgren *et al.*, 2017). WIsH (Galiez *et al.*, 2017) is another popular method for the association status prediction between sequences. It first trains an HMM using reference sequences, then a score will be assigned to each new sequence according to the trained HMM. Since the construction of HMM is not dependent on contig lengths, we can use it on any pair of contigs. So we first compared the performance difference among $d_{2}^{*}$, WIsH and ContigNet.

**Fig. 4.**
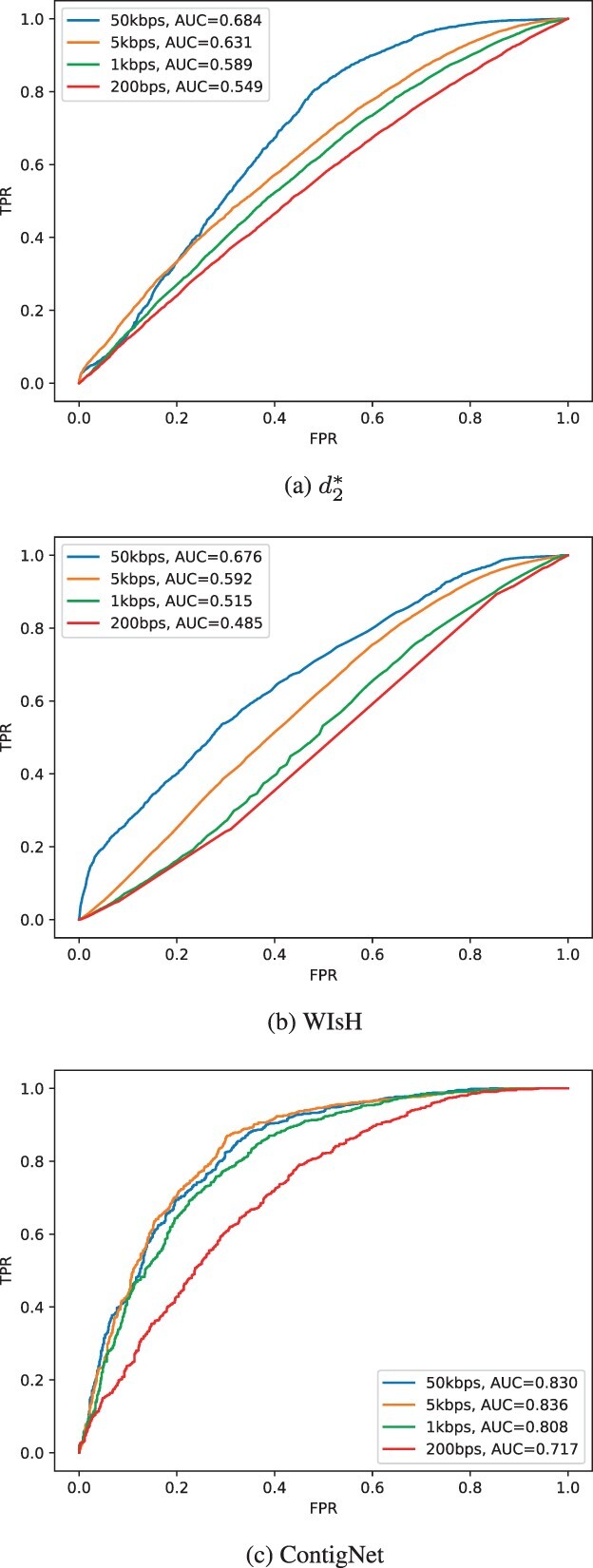
Performance comparison among $d_{2}^{*}$, WIsH and ContigNet. (**a**) The ROC curves of $d_{2}^{*}$ method on the validation set with contigs of different lengths. (**b**) The ROC curves of WIsH on the validation set with contigs of different lengths and (**c**) the ROC curves of ContigNet on validation set with contigs with different lengths

We used Afann (Tang *et al.*, 2019) to calculate the $d_{2}^{*}$ dissimilarity between phage and host pairs. The selection of negative phage–host pairs was the same as the one we used to train our model. To assess the performance of $d_{2}^{*}$ on contigs, we used 200 bps, 1 kbps, 5 kbps and 50 kbps as the contig lengths for both phage and host to sample from genomes. For experiments with contigs, for a given contig length *l*, we randomly sampled a contig from phage and host genomes from the validation set, respectively, and the sampling was repeated *k *=* *10 times. With the sampled contig pairs, we then evaluated the performance of $d_{2}^{*}$ using AUROC. The same approach was applied to WIsH and the AUROC score for each contig length was recorded. Similarly, we evaluated the performance of our trained model on contigs with different lengths using the same approach. To test our model, the contig pairs were sampled from the phage–host pairs in the validation set. The ROC curves for the three different methods with different contig lengths are shown in Figure 4.

Figure 4a shows that for $d_{2}^{*}$ the AUROC is markedly lower than that of ContigNet for virus-host contig association predictions. The AUROC score for $d_{2}^{*}$ is only 0.684 for contig pairs of 50 kbps and further drops to 0.589 for contig pairs of 1 kbps. Figure 4b depicts the performance of WIsH under different conditions, and we can see similar characteristic of the change of performance, for shorter contigs the performance drastically degraded. In comparison, as shown in Figure 4c, ContigNet achieves a much higher AUROC of over 0.808 for contig pairs of length above 1 kbps. Even for contig pairs of length 200 bps, the AUROC can be as high as 0.717.

There are several other tools for phage–host association prediction; however, they are not suitable for the question described in this study, as discussed in more detail in Section 4.

### 3.3 The performance of ContigNet increases with both viral and host contig lengths

Figure 4 shows that the performance of ContigNet increases with the contig length when the viral and host contigs are of the same length. ContigNet can be applied to predict phage–host associations even if the contigs are of different lengths and we next investigate how the performance of ContigNet changes with the viral or host contig lengths.

**Fig. 5.**
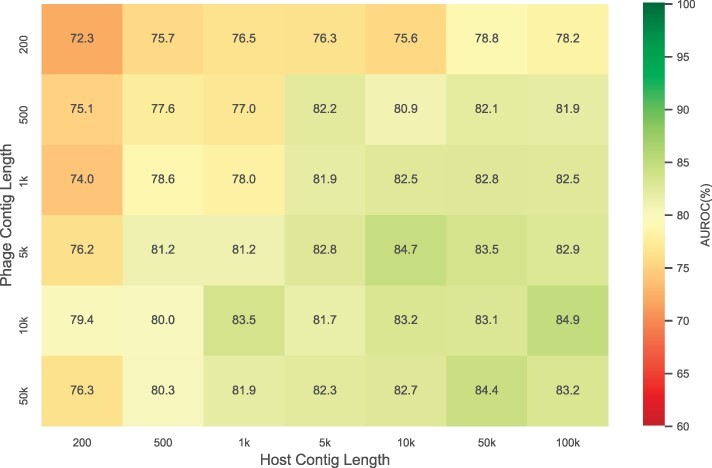
Heatmap of AUROCs for different phage and host contig lengths. The *x*-axis represents host contig lengths and the *y*-axis represents phage contig lengths

To assess the effect of different lengths of phage and host contigs on the performance of our trained model, we extended the contig length and evaluated the AUROC score of our model with contig length selected from 200 bps, 500 bps, 1 kbps, 5 kbps, 10 kbps and 50 kbps for phages and 200 bps, 500 bps, 1 kbps, 5 kbps, 10 kbps, 50 kbps and 100 kbps for hosts. The results are presented in Figure 5. The *x*-axis represents the host contig length, and *y*-axis marks the phage contig length. Figure 5 shows an apparent increasing trend of AUROC with respect to both phage and host contig lengths. When the phage contig length is about 1 kbps, the AUROC is stablized at around 0.82 when the host contig length is above 5 kbps. When the phage contig length is above 5 kbps, the AUROC is stablized at around 0.83 when the host contig length is above 10 kbps.

This observation can be intuitively explained by the fact that longer contigs contain more information that can be extracted by our network, resulting in a higher AUROC score.

### 3.4 Assessing the effects of different channels

For a DNA sequence, the information it contains can be classified into two granularities, base level and codon level. To incorporate these two granularities, a di-path model was utilized, i.e. two separate convolutional paths for each contig, one path for parsing base information and the other for codon information.

**Fig. 6.**
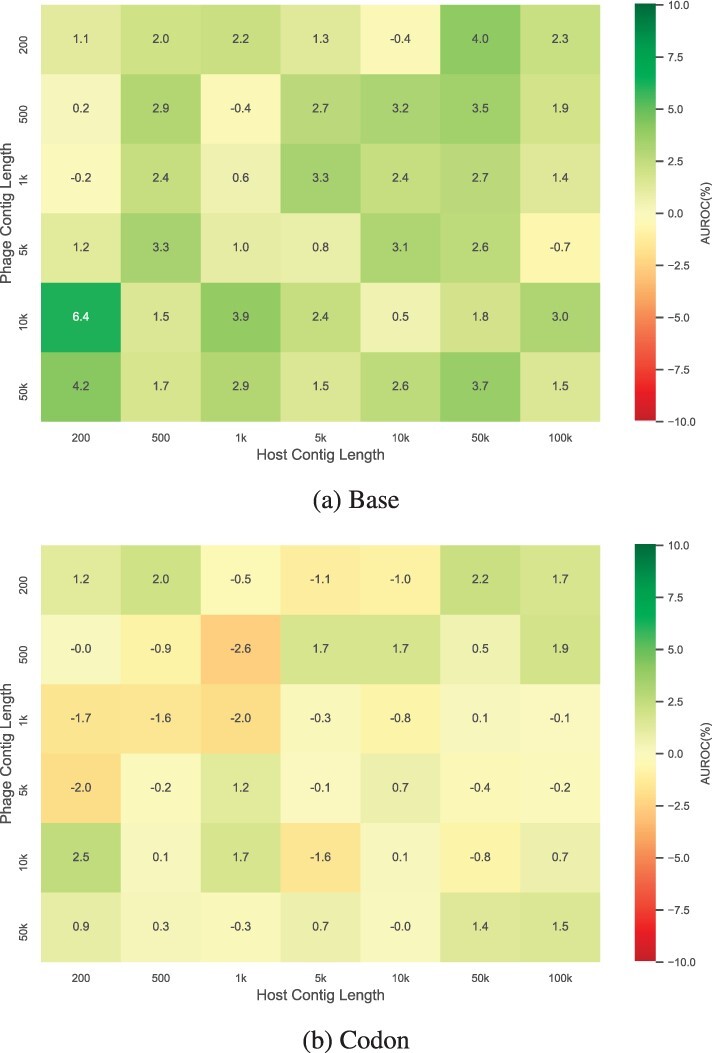
The AUROC value difference between ContigNet with di-path model and (**a**) base-path only model and (**b**) codon-path only model

Here we explore if the di-path model can improve the prediction results and the contributions of each path to the final result. To assess the contributions, we retrained our model by keeping only the base or codon path, assessed the performance of the single-path models, and compared their performance to our original di-path model. Figure 6 is the heatmap showing the difference of AUROC on corresponding contig lengths combinations between the di-path model and each single-path model. In the heatmap, deeper green means the di-path model performs better than the single path model, whilst red cell means the di-path model underperforms.

Figure 6 shows that the base-path model does not perform as well as the di-path and codon-path models, while the di-path model and the codon-path model perform similarly. We utilized the Wilcoxon signed-rank test to quantify the difference between models. For the base-path model and the di-path model, we got $p=2.41\times 10^{-8}$ with the alternative hypothesis that the AUROC of base-path model was smaller than that of the di-path model. This indicates that the di-path model performs significantly better than the base-path model. However, when comparing the codon-path model and di-path model, we got *p *=* *0.472 with two-sided alternative hypothesis, and thus we cannot reject the null hypothesis that the two models perform the same.

From the observation, we can conclude that the di-path model was not able to provide any significant advantage over using codon-path alone. Despite the aforementioned result, there was no apparent drawback of keeping both paths in the model compared to using only the codon path, and with multiple potential benefits of overparameterization in model convergence and generalization (Allen-Zhu *et al.*, 2019), we kept the di-path structure in our model.

### 3.5 Sequencing errors and chimeric contigs decrease the performance of ContigNet

**Fig. 7.**
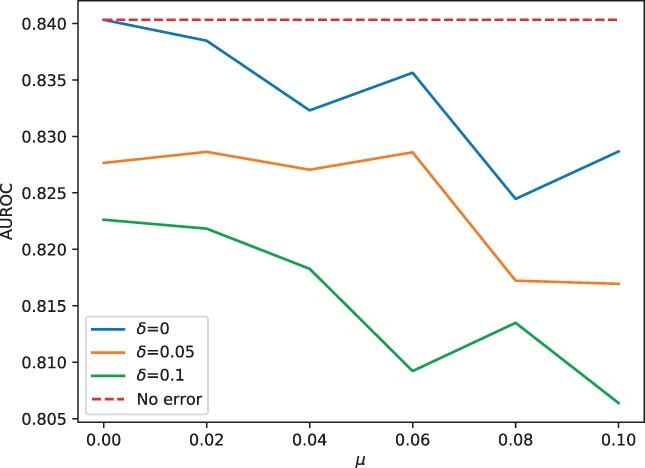
ContigNet performance with different levels of artificially introduced sequencing errors to the test set. The dash line stands for the baseline when no errors were introduced

**Fig. 8.**
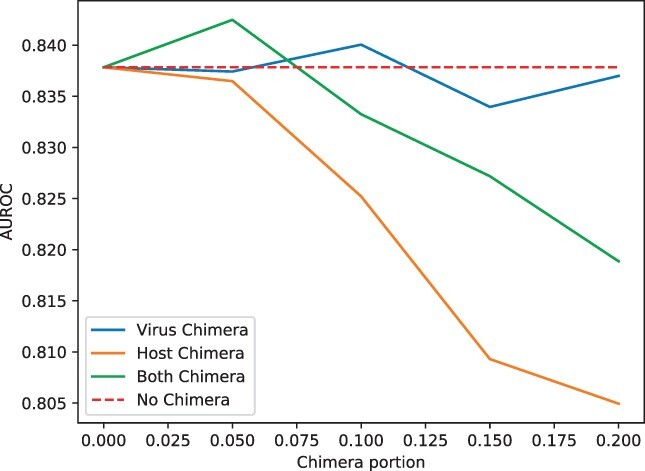
The performance of ContigNet with different levels of artificially introduced chimera for phage contigs only, host contigs only and both phage and host contig pairs. The dash line represents the baseline AUROC of ContigNet with no chimeras

Figures 7 and 8 show the performance of ContigNet trained with error-free training set and tested with validation set with artificially introduced errors. Figure 7 shows the change of performance with different levels of simulated sequencing error rates. The performance of ContigNet decreases with both substitution and insertion/deletion rates as expected. However, even with very high values of insertion/deletion rate δ=0.1 and substitution rate μ=0.1, ContigNet still maintains a high AUROC score of above 0.805, a slight decrease from 0.840 when no errors are introduced to the validation set.

Figure 8 shows the AUROC scores of ContigNet with different levels of artificial chimeras for phage contigs only, host contigs only and both phage and host contig pairs. The dash line shows the AUROC score of ContigNet for no chimeras as the baseline. Both the phage and host contig lengths were set at 5 kbps. The figure shows that ContigNet is robust to phage chimeras, with AUROC staying around that for ContigNet with no chimeras. The impact of host chimeras on ContigNet is higher than that for phage contigs. ContigNet still maintains AUROC above 0.805 when the fraction of sequence from others is below 0.20. For the different impacts between host chimeras and phage chimeras on ContigNet, it can possibly be attributed to the inherent diversity differences between phages and hosts. The phages are more diverse and are more likely to exchange genetic materials compared to hosts.

### 3.6 Performance of ContigNet on new datasets

To further validate that ContigNet works well for novel data, we tested ContigNet on two separate databases. The first database is the MGV catalogue (Nayfach *et al.*, 2021). The investigators used multiple viral-informative features, including the presence/absence of viral protein families, the presence of viral nucleotide signatures and multiple adjacent genes on the same strand (Nayfach *et al.*, 2021) to identify viral contigs based on 11 810 distinct human gut metagenomic samples. The viral genomes with completeness >50% were then selected from all identified viral genomes, and their hosts were identified using CRISPR-spacer matches and whole-genome alignment. Both methods require near exact matches and the predicted hosts for the viral genomes have a high specificity. We collected the phage–host pairs from the MGV database with hosts of the phages predicted at the genus level. A total of 77 348 phage–host pairs were identified. To remove phages with high similarity to phages in Virus-Host DB, we clustered the phage genomes from Virus-Host DB and MGV. The total number of phages were around 80 000. CD-Hit was not able to give a result in a reasonable time, due to not fully utilizing multi-threading. We applied MMseqs2 (Steinegger and Söding, 2017) with parameters local sequence identity with sequence identity threshold 95% and 50% alignment coverage for the shorter sequence, which were the same as we used when clustering the Virus-Host DB alone. Phage–host pairs from MGV with phage appeared in the same clusters as phages from Virus-Host DB were removed. Phage–host pairs with hosts under the same genus as hosts from VirHost DB were also removed. The resulting test set contains 39 916 phage–host pairs with 39 916 phages and 134 genera.

**Fig. 9.**
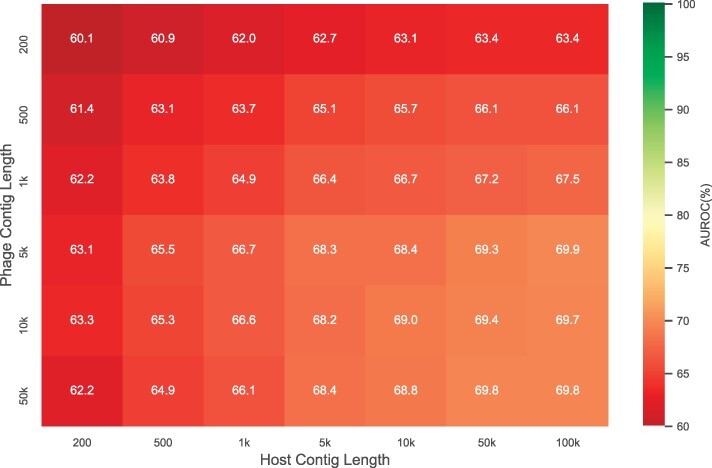
Heatmap of AUROC for different contig lengths on MGV dataset for model trained with Virus-Host DB. The *x*-axis represents host contig lengths and the *y*-axis represents phage contig lengths

The ContigNet model was trained on the entire Virus-Host DB, and tested on the MGV dataset. The AUROC results for different combinations of phage and host contig lengths are shown in Figure 9. As a baseline, we tested $d_{2}^{*}$ and WIsH on the MGV dataset with 50 kbps contigs. The selection of positive and negative pairs was the same as the aforementioned method. The AUROC score was 0.516 and 0.518, respectively. Shorter contigs were not tested because the AUROC score for longer contigs was already low. In comparison, the AUROC for ContigNet with 200 bps contigs pairs is 0.601. Compared to the result of $d_{2}^{*}$ and WIsH it is a significant improvement. With contig pairs of 50 kbps, the AUROC score is 0.698, a further improvement. The score converges as we further increase the phage and host contig lengths, with 0.698 for phage contig of 50 kbps and host contig of 100 kbps. The heatmap for different contig length combinations is shown in Figure 9. The AUROC scores are generally lower than that in Figure 5, probably due to sequencing errors and chimeric contigs.

**Fig. 10.**
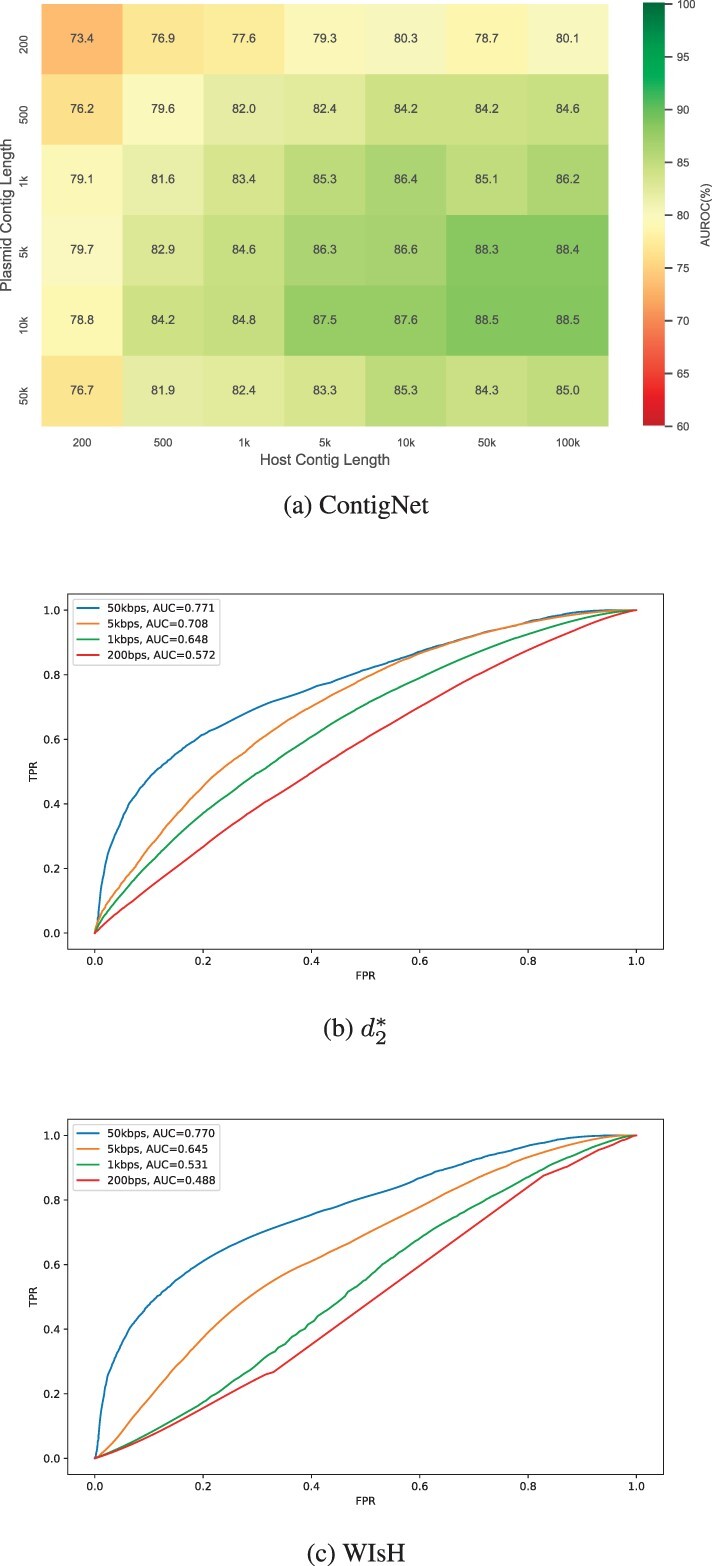
ContigNet can be used to predict plasmid-host associations with high accuracy. (**a**) Heatmap of AUROC for different contig lengths on plasmid dataset (PLSDB) for model trained with virus dataset (Virus-Host DB). The *x*-axis represents host contig lengths and the *y*-axis represents plasmid contig lengths. (**b**) The ROC curves of $d_{2}^{*}$ method on PLSDB for contigs with different lengths. (**c**) The ROC curves of WIsH on PLSDB for contigs with different lengths

The second database we used was the PLSDB dataset (Galata *et al.*, 2019). We wondered whether the ContigNet model we developed can be used to predict plasmid-host associations. For each plasmid entry in the PLSDB dataset, a corresponding host species was provided. To evaluate the performance of our model on PLSDB, we first trained our model on the entire Virus-Host DB. For each positive plasmid-host pair in PLSDB, a different host species were randomly selected to construct the negative plasmid-host pair. The AUROC was then calculated for different plasmid and host contig lengths ranging from 200 bps to 5 kbps, and the AUROC was reported for different contig length combinations. Because plasmid and phage are two different classes of MGEs, there were no overlaps between Virus-Host DB as training set and the PLSDB as testing set. Therefore, no redundancy removal steps were required. The results are shown in Figure 10a. The figure shows that the resulting AUROCs range from 0.734 to 0.85. For comparison, we calculated the AUROC scores when we used $d_{2}^{*}$ and WIsH to predict the association status when the contig lengths were 200 bps, 1 kbps 5 kbps and 50 kbps, respectively. The results are shown in Figure 10b and c, where the AUROC ranged from 0.489 to 0.771. By comparing the results, we can see that ContigNet has provided significant improvement even if the model was trained on a totally different dataset.

The performance of ContigNet on PLSDB seemed unexpected, considering the distinct lifestyle between plasmids and phages. However, from evolutional perspective, both MGEs depend on organelles of hosts to replicate and have to adapt to the internal environment of a particular host, including the bias of nucleotides, codons, di-codons etc. The experiment above proves that our model is able to capture this relationship between phages and hosts, and apply it to make plasmid-host association prediction.

### 3.7 Computational cost

The training of the model pre-loads the whole Virus-Host DB and corresponding hosts into the memory to reduce I/O overhead, so a machine with over 128GB memory is recommended. We used a workstation with AMD EPYC 7742 CPU, NVIDIA RTX 2080Ti GPU and it took <5 h for training, <1 h for testing.

## 4 Discussion

In this article, we present ContigNet, a deep learning-based method for predicting phage–host contig interactions, the first deep learning method for this task. We showed that ContigNet outperforms other *k*-mer-based method such as $d_{2}^{*}$ or Markov chain-based method, WIsH, for predicting phage–host contig associations.

Several state-of-the-art tools are available for phage–host association prediction, including PHP (Lu *et al.*, 2021), HoPhage (consisting of HoPhage-G and HoPhage-S) (Tan *et al.*, 2022), VPF-Class (Pons *et al.*, 2021), VHM-Net (Wang *et al.*, 2020), vHULK (Amgarten *et al.*, 2020), RaFAH (Coutinho *et al.*, 2021) and HostG (Shang and Sun, 2021). Each of these tools has its own limitations making it unsuitable for the question of predicting phage–host contig associations in natural metagenomic settings. For instance, HoPhage-G, VPF-Class, VHM-Net, vHULK and RaFAH are multiclass classifiers with fixed candidate host set and thus cannot be used for phage–host contig association predictions. PHP is another k-mer-based method and was shown to underperform WIsH when the viral contigs were shorter than 10 kbps (Lu *et al.*, 2021). HoPhage-S uses coding sequences to build HMM but short contigs may not contain coding sequences. HostG uses BLASTN matches to construct phage–host connections when building the graph and integrate new hosts into the graph. However, such matches can be scarce between contigs. Therefore, all aforementioned softwares are not suitable to predict the reference-independent contig-level phage–host associations.

**Fig. 11.**
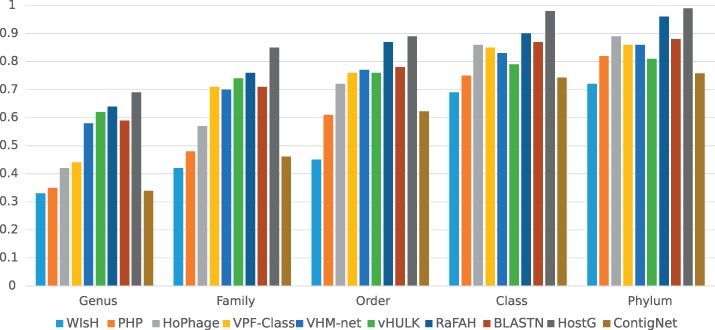
Host prediction accuracy for whole genomes from genus to phylum

**Fig. 12.**
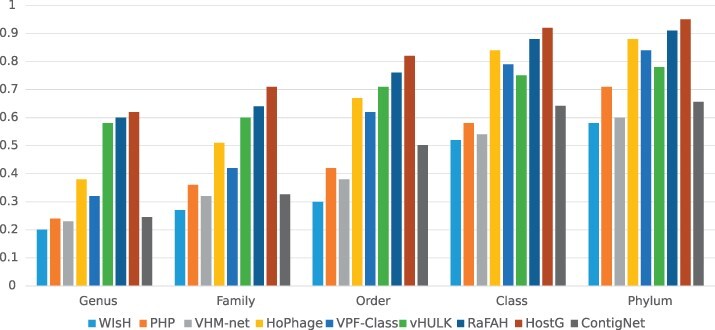
Host prediction accuracy for whole genomes without alignment results from genus to phylum

On the other hand, ContigNet can also take the whole phage and host genomes as input because it supports contigs with any length. We compared its performance with other methods on the whole genomes according to the experimental steps described in Shang and Sun (2021) by testing the methods on the whole dataset and the dataset with only phage–host pairs without alignment results. And the results are shown in Figures 11 and 12. From the figures, we can observe that ContigNet still outperforms WIsH. However, ContigNet underperforms other state-of-the-art approaches for phage–host genome associations that were optimized for such a purpose. Therefore, users are advised to use existing methods specialized for whole-genome association if the host whole genome is available.

With the incorporation of shared weights of convolutional paths between phage and host, we observed increased performance and generalizability. If we consider ContigNet as consisting of two components, the convolutional layers and the linear layers, the convolutional layers can be considered as a feature extractor and the linear layers can be considered as a classifier accepting the 512 features extracted from phage contig and host contigs, respectively. The applications of the extracted features are not restricted to the phage–host association status prediction itself, and the derived model can also be used for plasmid-host contig association predictions, a completely different problem. Our results show the great potential that our model can be adapted for much broader applications, either directly use the model for sequence feature extraction, or use our model as a foundation and use transfer learning to fine-tune the parameters for new problems. Therefore, in our software, we provide a feature extraction mode as well as the trained model so that other investigators can use features to train new models for different questions.

However, there are still more valuable topics to explore for this method in the future. Poor explainability has long been a shortcoming for deep learning-related methods in different applications, and this applies to our model too. In traditional methods, the features extracted from a sequence have clear meanings, for example, *k*-mer frequencies are the frequencies of oligonucleotides in the given sequence. Among the 512 features extracted from a sequence, 256 of them are from base information and 256 of them are from codon information, but there is no clear biological meaning for each individual feature. Therefore, an explainable feature extractor is a topic for future studies.

In conclusion, ContigNet showed a competitive performance on identifying the relationship between phage and host contigs. It can be a useful tool for biological researchers when studying novel metagenomic samples from diverse natural environments, particularly those ones poorly represented in genomic databases.

## Data availablity statement

The data underlying this article are available at https://github.com/tianqitang1/ContigNet

## Funding

This work was supported by the National Institutes of Health [R01GM120624, 1R01GM131407], the National Science Foundation [EF-2125142], the Simons Collaboration on Computational Biogeochemical Modeling of Marine Ecosystems/CBIOMES grant (ID: 549943) and the Gordon and Betty Moore Foundation (Grant Number: 3779).

*Conflict of Interest*: none declared.  
